# Descriptions of Adverse Drug Reactions Are Less Informative in Forums Than in the French Pharmacovigilance Database but Provide More Unexpected Reactions

**DOI:** 10.3389/fphar.2018.00439

**Published:** 2018-05-01

**Authors:** Pierre Karapetiantz, Florelle Bellet, Bissan Audeh, Jérémy Lardon, Damien Leprovost, Rim Aboukhamis, François Morlane-Hondère, Cyril Grouin, Anita Burgun, Sandrine Katsahian, Marie-Christine Jaulent, Marie-Noëlle Beyens, Agnès Lillo-Le Louët, Cédric Bousquet

**Affiliations:** ^1^Sorbonne Université, INSERM, Université Paris 13, Laboratoire d’Informatique Médicale et d’Ingénierie des Connaissances en e-Santé, Paris, France; ^2^Centre Régional de Pharmacovigilance, Centre Hospitalier Universitaire de Saint-Étienne, Hôpital Nord, Saint-Étienne, France; ^3^Université de Lyon, IMT Mines Saint-Etienne, Institut Henri Fayol, Département ISI, Université Jean Monnet, Institut d’Optique Graduate School, Centre National de la Recherche Scientifique, Laboratoire Hubert Curien, Saint-Étienne, France; ^4^Centre Régional de Pharmacovigilance, Hôpital Européen Georges-Pompidou, Assistance Publique – Hôpitaux de Paris, Paris, France; ^5^LIMSI, CNRS, Université Paris-Saclay, Orsay, France; ^6^INSERM UMRS1138 Centre de Recherche des Cordeliers, Paris, France; ^7^Département d’Informatique Médicale, Hôpital Européen Georges-Pompidou, Assistance Publique – Hôpitaux de Paris, Paris, France

**Keywords:** adverse drug reaction, adverse event, forum, internet, pharmacovigilance, social media, Web 2.0

## Abstract

**Background:** Social media have drawn attention for their potential use in Pharmacovigilance. Recent work showed that it is possible to extract information concerning adverse drug reactions (ADRs) from posts in social media. The main objective of the Vigi4MED project was to evaluate the relevance and quality of the information shared by patients on web forums about drug safety and its potential utility for pharmacovigilance.

**Methods:** After selecting websites of interest, we manually evaluated the relevance of the content of posts for pharmacovigilance related to six drugs (agomelatine, baclofen, duloxetine, exenatide, strontium ranelate, and tetrazepam). We compared forums to the French Pharmacovigilance Database (FPVD) to (1) evaluate whether they contained relevant information to characterize a pharmacovigilance case report (patient’s age and sex; treatment indication, dose and duration; time-to-onset (TTO) and outcome of the ADR, and drug dechallenge and rechallenge) and (2) perform impact analysis (nature, seriousness, unexpectedness, and outcome of the ADR).

**Results:** The cases in the FPVD were significantly more informative than posts in forums for patient description (age, sex), treatment description (dose, duration, TTO), and outcome of the ADR, but the indication for the treatment was more often found in forums. Cases were more often serious in the FPVD than in forums (46% vs. 4%), but forums more often contained an unexpected ADR than the FPVD (24% vs. 17%). Moreover, 197 unexpected ADRs identified in forums were absent from the FPVD and the distribution of the MedDRA System Organ Classes (SOCs) was different between the two data sources.

**Discussion:** This study is the first to evaluate if patients’ posts may qualify as potential and informative case reports that should be stored in a pharmacovigilance database in the same way as case reports submitted by health professionals. The posts were less informative (except for the indication) and focused on less serious ADRs than the FPVD cases, but more unexpected ADRs were presented in forums than in the FPVD and their SOCs were different. Thus, web forums should be considered as a secondary, but complementary source for pharmacovigilance.

## Introduction

Pharmacovigilance, defined as “the science and activities relating to the detection, assessment, understanding, and prevention of adverse effects or any other drug-related problem” (World Health Organization, 2018), depends mainly on spontaneous reporting (Pal et al., 2013). ADR reporting was first limited to healthcare professionals, but has progressively opened to patients using dedicated platforms in many countries in the last decade (Margraff and Bertram, 2014). Several studies have demonstrated the value of the information given by patients on ADRs (Al Dweik et al., 2017; Rolfes et al., 2017). However, under-reporting and incomplete data are still major limitations for pharmacovigilance (Gonzalez-Gonzalez et al., 2013; Varallo et al., 2014).

In the age of Web 2.0, researchers have started to focus on exploring social media as a complementary source for pharmacovigilance (Sarker et al., 2015; Tricco et al., 2017). Forums, social media and microblogging platforms allow patients to ask medical questions or share experiences with others in the same condition particularly for chronic diseases. In France, 48.5% of Web users aged from 15 to 30 years searched for health information online in 2010 (Beck et al., 2014). A national American survey reported a rate that reached 72% for all ages in 2013. In the same survey, 18% of users had consulted online reviews of specific drugs or medical treatments (Pew Research Center, 2013).

The amount of potential information in web forums and its immediate availability provide excellent opportunities for pro-active surveillance of ADRs. Four reviews were recently published on this topic (Golder et al., 2015; Lardon et al., 2015; Sarker et al., 2015; Sloane et al., 2015). They confirmed that mining social media could lead to the identification of ADRs, including unexpected ones. However, they highlighted that several technical challenges related to terminology, traceability, or reliability of the extracted data are yet to be addressed. They also emphasized the heterogeneity of the included studies concerning methodological quality and risk of bias.

Some authors used the term “ADRs,” instead of “AEs” (adverse events), to refer to co-occurrences of drugs and adverse experiences in the comments of patients, without validation of causal relationships (risk of false positives and misinformation). Only one study (Kheloufi et al., 2017a) considered all the criteria required to assess causality, but it was only applied to a small number of reports, extracted from a specialized online drug reporting website (MeaMedica).

Traditional pharmacovigilance is based on spontaneous pharmacovigilance report including at least four elements: a patient, a suspected drug, an AE, and a reporter (Edwards et al., 1990; EMA, 2017). The report is evaluated by verifying its completeness regarding data on the patient and the drug and the description of the effect, with particular attention to all information needed to assess causality, such as treatment dates, TTO, drug indication, patient characteristics, and outcome. If the case is validated, the report is registered in a dedicated pharmacovigilance database with a causality assessment. Finally, the pharmacovigilance team evaluates the interest of the case as a potential signal and, if needed, transmits it immediately to the competent authority (competent national authority or marketing authorization holder). In the perspective of using social media for pharmacovigilance, we postulated that posts could be managed as spontaneous case reports.

The Vigi4MED project (pharmacovigilance in web forums) aimed to evaluate whether social media can be a valuable source of information on drug safety to provide health authorities with a source of information that complements standard pharmacovigilance data^1^. Posts should be processed similarly to case reports to follow the usual pharmacovigilance workflow. This includes identifying a patient (sex and age), a reporter (the patient or a relative), a suspected drug, and at least one adverse effect (EMA, 2017).

The objective of our study was to evaluate the potential of social media to provide useful and reliable information for pharmacovigilance. Within the Vigi4MED project, we first assessed data concerning drug safety from forums (completeness, quality, ability to perform impact analysis), and then compared these indicators with those coded in the FPVD for case reports with the same drug, over the same period of time, to evaluate the added value of this new source of information.

## Materials and Methods

### Summary of the Vigi4MED Project

**FIGURE 1:**
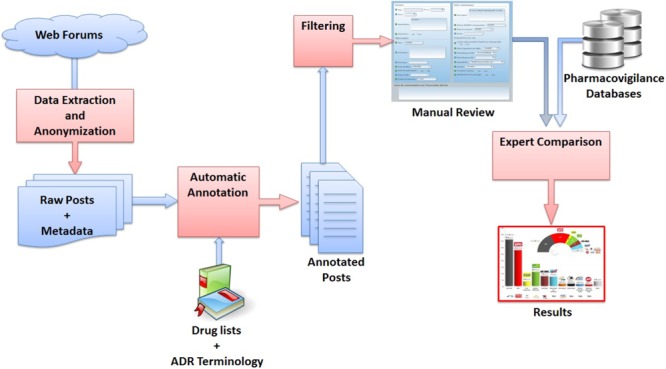
General overview the Vigi4Med project.

The Vigi4MED project included a total of seven partners: five research units specialized in computer science (medical informatics, NLP, and semantic web) and two pharmacovigilance centers. It was conducted in four main steps: (1) data extraction and anonymization from health web forums that are potentially interesting for drug safety, using automatic methods; (2) automatic detection and annotation of co-occurrences of drugs and AEs in the corpus using advanced NLP techniques and resources; (3) filtering, i.e., post selection; and (4) expert comparison between posts in web forums and reports in the FPVD. This comparison relied on evaluating the usefulness and reliability of the data for drug safety monitoring. This evaluation was performed using a dedicated web interface and causality assessment of selected patient posts containing potential ADRs, using a methodology similar to that used for spontaneous reports. A general overview of the project is presented in **Figure 1**.

We generated a dataset of over 60 million posts extracted from 22 French medical forums – one website could host several forums – from 01/01/2000 to 07/03/2015, using the open source tool Vigi4Med Scraper (Audeh et al., 2017), which was developed for the Vigi4Med project. NLP techniques were then applied to these posts (Morlane-Hondère et al., 2016) to identify both drugs (drug name and posology) and events (disorders, signs, or symptoms) along with their localization (anatomical region). The procedure to find the associations between drugs and events was implemented but did not provide the expected result due to the multiple ways that causal relations are expressed in social media. Thus, AEs rather than ADRs were considered. Our material consisted of 55,350,564 drug/AE pairs in 6,569,555 posts.

Among this huge amount of data, we selected six drugs to perform a retrospective evaluation: tetrazepam as a drug withdrawn or suspended from the market for pharmacovigilance reasons, baclofen that has been the subject of media coverage, and four drugs that have been recently marketed or monitored with a risk management plan (agomelatine, duloxetine, exenatide) or that are under reinforced monitoring (strontium ranelate).

### Selection of Study Periods and Websites

For each drug, we compared data in forums and the FPVD within the same period, from first use in France to the end of the study, for four drugs (agomelatine, duloxetine, exenatide and strontium ranelate). For the last two drugs studied (baclofen and tetrazepam), we selected a 2-year period surrounding safety issues:

• Agomelatine: From 05/28/2010 (commercialization in France) to 06/17/2015 (end of post extraction)
• Baclofen: From 01/01/2013 to 12/31/2014 (2-year period around 03/17/2014, the date of the TRU allowing its prescription to treat alcohol dependence)
• Duloxetine: From 11/26/2007 (commercialization in France) to 06/17/2015 (end of post extraction)
• Exenatide: From 04/03/2008 (commercialization in France) to 06/17/2015 (end of post extraction)
• Ranelate strontium: From 01/01/2004 (commercialization in Europe) to 17/06/2015 (end of post extraction)
• Tetrazepam: From 01/01/2012 to 31/12/2013 (2-year period around the evaluation of a safety signal and drug withdrawal).

The Vigi4MED project targeted French health-related websites for the general public. We identified potential relevant websites by either performing a simple search on Google using the terms “drug” AND “adverse drug reaction” OR “adverse event” AND “forum” (translated from French) or searching from the list of health websites certified by the HON Foundation, in collaboration with the French National Health Authority (*Haute Autorité de Santé* in French, also abbreviated as HAS), available at http://services.hon.ch/cgi-bin/HONcode/browse_f.pl?RC=FR&t=name.

After exploration, websites were excluded if they were hosted outside of France, did not contain a discussion board or a space to share experiences, contained less than 10 patient contributions, or were reserved for health professionals and thus did not contain patient comments.

### Evaluation of the Posts

For each evaluated drug, and according to the volume of data for each of them, several approaches were considered to select the candidate posts to describe an ADR (**Table 1**). The first consisted of reviewing all posts containing the drug of interest and a potential ADR. When the number of posts was too high for their available resources (workforce and time), pharmacovigilance experts performed random sampling or manual selection of posts focused on a specific ADR and/or a misuse situation. The last approach consisted of selecting posts after application of the PRR algorithm (Evans et al., 2001) (with the traditional decision rule: at least three cases, PRR of at least two, and Chi-squared of at least four) on the 55,350,564 drug/AE pairs to limit the noise due to false positives and exclude AEs corresponding to the indication or complications of the treated disease.

**Table 1:** Various approaches used for post selection before evaluation.

	Type of selection				Comments
	All posts	Random sampling	Focus on an AE and/or misuse situation	PRR^1^	
Agomelatine				✓	Exclusion of the ADRs linked to indication: « depressed mood », « depression », « psychological trauma », etc.
Baclofen			✓		ADR: depressive state, Misuse situations: use outside marketing authorization (MA)/TRU (bulimia nervosa, toxicology weaning, parkinsonian tremor, etc.)
Duloxetine		✓			Pilot study
Exenatide				✓	Exclusion of the AEs linked to indication: « type 2 diabetes mellitus », « overweight », « polyneuropathy », etc.
Strontium ranelate	✓				
Tetrazepam	✓				

*^*1*^PRR, proportional reporting ratio.*

The extracted posts were reviewed using a dedicated web interface implemented for this study (DL). The posts were loaded in a random order. Thus, for random sampling, we considered the reviewed posts to correspond to a random sample when the review was halted. The reviews were performed by trained pharmacists (RA, FB) and in case of discrepancies, individual cases were assessed by senior pharmacovigilance professionals (ALL, MNB). If the post described an ADR, the following information was included in our analysis and stored in the database *via* the web interface:

• Patient data: Age, sex, medical history, and pregnancy status
• Treatment data: Drug, indication, dose, dates of starting and stopping medication, medication stopped or not
• The ADR: Medical Dictionary for Regulatory Activities (MedDRA) term (as used in the FPVD) coded using PTs and SOCs^2^, start date, duration
• The evaluation of the case:
  ◦ Compatibility of the TTO, dechallenge, rechallenge
  ◦ Drug causality
  ◦ Case seriousness: At least one ADR which results in death, is life-threatening, requires patient hospitalization or prolongation of an existing one, results in persistent or significant disability or incapacity, is a congenital anomaly/birth defect or is another important medical event
  ◦ Expectedness: ADR labeled in the SPCs

For each drug, posts were validated as potential case reports if they were posted during the study period and could be considered as pharmacovigilance cases, i.e., they at least contained the following elements (EMA, 2017): a reporter (the post author), a patient (the post author or a relative), and an ADR which could be imputable to the drug of interest and corresponded to a code in the MedDRA terminology (PT and SOC levels).

Posts were not validated as potential case reports if they were: undated, posted by a patient mentioning that he was not residing in France, not related to the experience of the post author or a relative, or identified as a duplicate of another already validated post.

Examples of non-validated posts are:

• False positive due to the NLP tool considering an indication as an AE
• An answer to or a comment about a post describing the experience of an ADR
• Description of an ADR that the post author heard via a third-party (“My neighbor told me that….,” “I’ve read that…”)
• A question about a potential ADR “Have you experienced the effect Y when taking X?”

### Data Extraction From the FPVD

The FPVD was created in 1985 to centralize anonymized cases of ADRs reported in France and collected by the 31 French Pharmacovigilance Centers under the responsibility of the French National Agency for the Safety of Medicines and Health Products (ANSM^3^).

The cases can be reported by health professionals and, since 2011, patients and patient associations. By June 17, 2015, when we completed the extraction, the FPVD contained 582,193 reports. FPVD cases are evaluated using the French causality assessment method. FPVD extraction requests were performed by the ANSM. The request criteria were:

• Investigated period: Same study periods for the forums
• Drugs: Tetrazepam, baclofen, duloxetine, strontium ranelate, exenatide, or agomelatine coded as suspect
• All ADRs
• Seriousness: Serious and non-serious.

Information concerning dechallenge and rechallenge extracted from the FPVD was only available for agomelatine and exenatide. The posts which concerned these two drugs are designated “AgEx-posts.” TTO was only available for agomelatine, duloxetine, and exenatide and the data were provided as Excel files by the ANSM to allow statistical analysis.

### Comparison of Patient Forums and the FPVD

We compared the informativeness of the two data sources (i.e., presence/absence of some information in a case/post) between forums and case reports in the FPVD mentioning the same drug for the following variables:

– Patient profile: Age (numeric and qualitative) and sex
– Treatment: Indication, dose, and duration
– ADR: TTO and outcome.

The presence of information concerning the outcome of the ADR after withdrawal of the drug in forums was defined as a positive or negative dechallenge.

We then compared forums to the FPVD for the following variables:

– Patient profile: Age (numeric) and sex
– Drug: Dechallenge and rechallenge
– ADR profile: Nature (SOCs), seriousness, unexpectedness, and outcome.

Quantitative variables (age) were compared using the Student test or Wilcoxon test, when the Student test was not applicable. For qualitative (categorical) variables, we performed the Chi-squared test or Fisher’s exact test. Tests were considered to be significant for a *p*-value <0.05.

We compared the SOC distribution between the two sources by considering all SOCs with a frequency >10 for each source and grouped the other SOCs together.

Analyzing the sum of the frequencies of the ADRs could be a source of bias, as the number of posts/cases differed between drugs. Thus, we considered a second total (“adjusted total”), which is the mean of the rates. To calculate the rate, posts with missing information (NA) were not considered. The “adjusted age” could not be statistically compared (the adjusted age corresponding to the mean of the age means and a whole distribution being required to perform a comparison).

Performed tests that were significant (*p* < 0.05) are designated by bold numbers followed by “^∗^” in the tables.

## Results

### General Findings

We found 2,521 cases in the FPVD with an average of 2.1 ADRs per case (5,262 ADRs in total), of which only 4.8% were reported by patients (**Table 2**). We reviewed 5,149 posts. Among them, 24.9% (1,284) were validated and 3,001 ADRs were found (2.3 ADRs/post on average). These 1,284 posts came from only eight of the 20 websites selected (**Table 3**). Most came from baclofene.fr (57.8%), but doctissimo.fr was the principal source for the six drugs (64.0%), when adjusting the frequency of cases per drug. Posts from the remaining 12 websites were not included because either they did not contain information about any of the six chosen drugs, posts containing study drugs in these websites were not validated as describing a potential case report, or were not reviewed after posts selection.

**Table 2:** Reporters of the cases in the FPVD.

Source	*N*	%	Adjusted %
Medical specialist	1,699	67.6%	65.1%
Pharmacist	386	15.3%	16.6%
General practitioner	281	11.2%	11.4%
Non-health professional	121	4.8%	5.5%
Other professional	28	1.1%	1.5%
NA	6	NA	NA
Total	2,521	100%	100%

**Table 3:** Validated posts sources.

Website	*N*	%	Adjusted %
baclofen.fr	742	57.8%	16.4%
doctissimo.fr	454	35.4%	64.0%
atoute.org	34	2.6%	5.1%
esante.fr	29	2.3%	10%
vulgaris-medical.com	12	0.9%	1.3%
onmeda.fr	6	0.5%	1.9%
sante.journaldesfemmes.com	5	0.4%	0.9%
allodocteurs.fr	2	0.2%	0.4%
Total	1,284	100%	100%

### Comparison of the Informativeness

Although the average number of ADRs was similar for both sources, the informativeness of the cases from the forums was significantly less than that of the FPVD cases concerning patient information (10.1% vs. 94.1% for age and 49.8% vs. 99.5% for sex, when adjusted), most treatment information (16.0% vs. 49.4% for the dose and 38.4% vs. 61.5% for the duration, when adjusted), and AEs (23.9% vs. 68.8% for the TTO and 15.1% vs. 85.3% for the outcome, when adjusted) (**Table 4**). Only the indication of the treatment was globally more frequently known in forums than in the FPVD (57.8% vs. 37.8%, when adjusted) (**Table 4**).

**Table 4:** Comparison of the age and sex of patients and informativeness of the cases/posts.

			Total		Adjusted total	
			FPVD	Forums	FPVD	Forums
			*N* = 2,521	*N* = 1,284	*N* = 2,521	*N* = 1,284
Patient	Age (numeric)	Informativeness	93.3%^∗^	4.8%	94.1%^∗^	10.1%
		Mean (SD)	57.3 (17.4)^∗^	44.4 (14.4)	57.3 (NA)	40.4 (NA)
		Median	57.0	44.0	57.2	39.4
		(Q1–Q3)	(45.0–70.0)	(36.0–55.0)	(NA)	(NA)
		NA	172	1,223	172	1,223
	Age (class)	Informativeness	93.3%^∗^	8.9%	94.1%^∗^	14.9%
	Sex	Informativeness	99.5%^∗^	47.7%	99.5%^∗^	49.8%
		Female	1,550 (61.8%)	406 (66.2%)^∗^	62.6%	75.7%^∗^
		Male	958 (38.2%)	207 (33.8%)	36.4%	24.3%
		NA	13	671	13	671
Treatment	Indication	Informativeness	37.1%	60.7%^∗^	37.8%	57.8%^∗^
	Dose	Informativeness	45.0%^∗^	23.4%	49.4%^∗^	16.0%
	Duration	Informativeness	54.7%^∗^	22.6%	61.5%^∗^	38.4%
AE	TTO^1^	Informativeness	83.5%^∗^	19.9%	68.8%^∗^	23.9%
	Outcome	Informativeness	82.3%^∗^	6.4%	85.3%^∗^	15.1%

*^*1*^TTO, time-to-onset, only available for agomelatine, duloxetine, and exenatide, ^∗^*p* < 0.05.*

### Comparison of Patient Profiles

Patients in forums were younger than those in the FPVD (mean age of 44.4 vs. 57.3 years – **Table 4**). However, comparison of the age of the patients between these two data sources may be of little relevance, as the information was known for only 4.8% of the patients in the forums.

Patients were mostly women for both data sources, but the proportion of women was higher in forums than in the FPVD (75.7% vs. 62.6% when adjusted, *p* < 0.05).

Only two pregnancies were identified in forums vs. 17 in the FPVD (note that this information was only available for agomelatine, duloxetine, and exenatide in the FPVD extract we used).

### Comparison of the ADR Profiles

There were significantly^4^ more serious cases in the FPVD than in the forums. Indeed, 1,150 serious cases were found in the FPVD, whereas only 27 serious cases were found in the forums, which corresponds to 45.6% in the FPVD vs. 2.1% in the forums, when the values were not adjusted for the number of posts/cases and 45.6% in the FPVD vs. 4.2% in the forums when adjusted. Thus, more deaths were identified in the FPVD than in the forums (61 vs. 3, i.e., 2.5% vs. 0.2%).

The ADRs identified in the forums represented less SOCs than in the FPVD (24 vs. 26). The distributions of the SOCs were significantly different between the FPVD and the forums (**Table 5**). The most frequent SOC in forums was psychiatric disorders (whether the distribution was adjusted or not – 23.6% and 30.9%, respectively), whereas the most frequent SOC in the FPVD was nervous system disorders (19.2%), when not adjusted, and gastrointestinal disorders (15.8%), when adjusted.

**Table 5:** Distribution of the SOCs in the forums and the FPVD.

SOC^1^	Total					
	FPVD		Forums		Adjusted %	
	*N*	%	*N*	%	FPVD	Forums
Psychiatric disorders	705	13.4%	926	30.9%	11.29%	23.6%
Nervous system disorders	1,008	19.2%	521	17.4%	15.57%	16.5%
Gastrointestinal disorders	704	13.4%	306	10.2%	15.79%	15.6%
General disorders and administration site conditions	374	7.1%	415	13.8%	6.96%	18.2%
Skin and subcutaneous tissue disorders	638	12.1%	102	3.4%	15.49%	3.5%
Investigations	203	3.9%	178	5.9%	4.66%	5.6%
Metabolism and nutrition disorders	187	3.6%	152	5.1%	3.32%	4.7%
Musculoskeletal and connective tissue disorders	202	3.8%	97	3.2%	3.22%	4.3%
Injury, poisoning and procedural complications	191	3.6%	98	3.3%	3.60%	3.0%
Respiratory, thoracic and mediastinal disorders	179	3.40%	53	1.8%	3.21%	0.5%
Vascular disorders	152	2.89%	15	0.5%	2.46%	0.6%
Ear and labyrinth disorders	92	1.75%	50	1.7%	1.52%	1.0%
Hepatobiliary disorders	137	2.60%	3	0.1%	3.11%	0.4%
Eye disorders	91	1.73%	41	1.4%	1.88%	1.1%
Renal and urinary disorders	102	1.94%	9	0.3%	2.04%	0.1%
Cardiac disorders	78	1.48%	15	0.5%	1.26%	0.7%
Blood and lymphatic system disorders	86	1.63%	0	0.0%	1.87%	0.0%
Reproductive system and breast disorders	36	0.7%	16	0.5%	0.5%	0.7%
Infections and infestations	27	0.5%	2	0.1%	0.8%	0.0%
Endocrine disorders	18	0.3%	0	0.0%	0.2%	0.0%
Congenital, familial and genetic disorders	14	0.3%	0	0.0%	0.3%	0.0%
Immune system disorders	11	0.2%	0	0.0%	0.3%	0.0%
Neoplasms benign, malignant and unspecified (including cysts and polyps)	11	0.2%	0	0.0%	0.5%	0.0%
Social circumstances	4	0.1%	2	0.1%	0.0%	0.0%
Pregnancy, puerperium and perinatal conditions	5	0.1%	0	0.0%	0.1%	0.0%
Product issues	4	0.1%	0	0.0%	0.04%	0.0%
Surgical and medical procedures	3	0.1%	0	0.0%	0.1%	0.0%
Total	5,262	100.0%	3,001	100.0%	100.0%	100.0%

*^*1*^SOC, system organ class.*

Most of the reported cases were expected ADRs for both data sources. Nevertheless, there were significantly more cases reporting unexpected ADRs in the forums than in the FPVD: 403 cases against 343, corresponding to 24.2% in the forums vs. 17.1% in the FPVD, when adjusted, and 31.4% vs. 14.7%, when not.

We compared ADRs in cases classified as unexpected in the forums to those in the FPVD for the six drugs. In total, 193 ADRs from unexpected cases present in the forums were absent from the FPVD, of which seven were serious (“Alcoholism,” “Crying,” “Impulse-control disorder,” “Fatigue,” “Irritability,” “Pain in extremity,” and “Breast enlargement”).

### Comparison of the ADR Outcomes

Use of the drug was stopped significantly more often in cases from the FPVD than those from forum posts (84.2% vs. 15.7% for all posts and 28.0% for AgEx-posts). Data concerning favorable outcomes after the drug was stopped were rarely available from the forums (6.4% for all posts, 16.6% for AgEx-posts) but a favorable outcome was not observed at a significantly higher frequency in the FPVD than the forums (90.3% vs. 85.4% for all posts and 81.0% for AgEx-posts). These results are described in **Table 6**.

**Table 6:** Comparison of the dechallenge between the sources.

Dechallenge	Medication stopped	Forums	AgEx-posts^1^	FPVD
Medication stopped	Yes	176 (15.7%)	56 (28.0%)	309 (84.2%)^∗^
	No	945 (84.3%)	144 (72.0%)	58 (15.8%)
	NA	163	5	36
	Total	1,284	205	403

Improvement	Yes	70 (85.4%)	34 (81.0%)	251 (90.3%)
	No	12 (14.6%)	8 (19.0%)	27 (9.7%)
	NA	1,202	163	125
	Total	1,284	205	403

*^*1*^AgEx, only posts concerning agomelatine or exenatide, ^∗^*p* < 0.05.*

## Discussion

This retrospective study, based on the analysis of data available for six selected drugs (agomelatine, baclofen, duloxetine, exenatide, ranelate strontium, and tetrazepam), allowed us to identify 1,284 pharmacovigilance cases, among 5,149 posts extracted from French language forums reviewed by pharmacovigilance experts. Thus, Web forums contain posts concerning drugs, adverse effects, and their potential association; they may thus qualify as a possible source of ADRs.

Comparison with the FPVD data showed that the average number of ADRs per post/case was similar for both sources. However, the forums were significantly less informative than the FPVD concerning patient information, most treatment information, and outcomes of the AEs. Only the indication of the treatment was significantly more frequently known in the forums than FPVD.

Interest in the use of social media for pharmacovigilance has been growing for several years and previous studies have shown the feasibility of extracting information on drugs and related ADRs from Web forums (Schröder et al., 2007; Moncrieff et al., 2009; Leaman et al., 2010; Butt et al., 2012; Mao et al., 2013; Wu et al., 2013; Yates et al., 2013; Abou Taam et al., 2014; Ferrara et al., 2014; Pages et al., 2014; Sampathkumar et al., 2014; Vaughan Sarrazin et al., 2014; Nikfarjam et al., 2015; Yang et al., 2015; Korkontzelos et al., 2016; Cocos et al., 2017; Kheloufi et al., 2017a; Lee et al., 2017; Piccinni et al., 2017; Tutubalina and Nikolenko, 2017), but the poor informational content of the identified cases still presents a challenge. Kheloufi et al. (2017a) recently published a study which used 16 criteria to assess the quality of 72 posts mentioning ADRs related to statins. However, their results are not easily transposable to forums in general as the website they used – *MeaMedica* – is a web platform in which patients share their experiences through a form containing information concerning their profile (age and sex) and the drug (indication, dose, duration …). Although patients consistently provided information concerning their age and sex, they were less forthcoming concerning the indication, dose, duration, TTO, and outcome of the AE, for which the response rate was less than 50% (24%, 17%, 18%, 31%, and 39%, respectively).

Only 4.8% of the FPVD cases we studied were declared by a non-professional (5.5% when adjusted). Thus, it seemed natural that the information in forums and the FPVD would differ. In addition, the lack of information of patient posts relative to that of cases in the FPVD was predictable without considering the status of the reporter. Indeed, information extracted from web forums corresponds to “raw data,” whereas that contained in the FPVD may have been completed following a demand to the reporter: their informativeness would not thus reflect that of the first report once in the FPVD. Lagneau et al. (2017) concluded that there was no significant difference in the informativeness between the initial declaration of patients and health professionals. Moreover, Kheloufi et al. (2017b) concluded in their 2017 study that the informativeness of patient declarations via the classic system could be significantly increased by contacting the reporter. Otherwise, patients may have distilled information of interest from several posts within a discussion topic. Pages et al. (2014) proposed to explore the same discussion topics over two periods, separated by 3 months, to obtain the maximum information concerning the outcome of the reported ADRs. However, it was not possible to relate posts published by the same patient in the context of our study.

Patients for both data sources were mostly women. Based on the 5% (10% when adjusted) of posts containing age information, the patients in forums were also younger than those in the FPVD (mean age of 44 vs. 57 years; adjusted age of 40 vs. 57 years). Our results are concordant with published studies: women and young patients are more willing to publish posts in social media (Pew Research Center, 2013; Golder et al., 2015).

The distribution of the SOCs was significantly different (*p* < 0.05) between the two sources and they seemed to be more diverse in the FPVD than the forums: the three most frequent SOCs represented 47% of those in the FPVD vs. 58% in the forums. The collaborative aspect of web forums could have biased the estimation of the frequency of certain ADRs reported in the forums: a first post could lead to several others reporting the same type of ADR for the discussion topic.

Furthermore, the proportion of non-serious cases reported in web forums was significantly higher than those reported in the FPVD (95.8% vs. 54.4%, when adjusted). Although ADRs reported in forums were more subjective than those in the FPVD and most were expected (76% when adjusted), forums contained significantly more cases with an unexpected AE than the FPVD (24.2% vs. 17.1%, when adjusted). A total of 193 ADRs from unexpected cases present in the forums were absent from the FPVD. These results confirm those of the medical literature (Abou Taam et al., 2014; Pages et al., 2014; Golder et al., 2015; Lardon et al., 2015).

Several cases were excluded from our study, as they were posted by patients from francophone countries (e.g., Belgium, Quebec) in which the evaluated drugs could be prescribed for indications other than those approved in France. This could have led to a bias in the evaluation of misuse. This issue has already been raised by Coloma et al. (2015).

The protocol considered the same study period, as well as alignment of the variables, to increase the comparability between the two data sources.

We considered cases reported in forums as ADRs, instead of AEs, and thus accounted for the possible presence of a causality link with the cited drug. Such causality was evaluated for a limited amount of data, whereas cases from the FPVD are generally considered to be causal after rigorous analysis, principally of electronic health records, ensuring the elimination of differential diagnoses.

Another limitation of this study was the mismatch between medical and patient vocabularies, as well as potential differences in the manner in which pharmacovigilance professionals aligned AE terms in posts to MedDRA terminology, in particular for comparisons between ADRs extracted from social media and those registered in the FPVD.

We cannot exclude possible over-estimation of the ADRs found in forums, although measures were taken to limit such bias. Indeed, the same patient could have reported his experience in several discussion topics on different web forums or websites with different usernames. Moreover, internet users could have reported the experience of another user or cited his own post. Finally, we chose to evaluate drugs mostly used for chronic diseases, making them more likely to be found in web forums. Moreover, the small number of drugs studied – six drugs, of which two are antidepressants – and their analysis together, raises questions concerning the generalizability of our results. However, the results were consistent for all the studied drugs. This consistence allows us to provide a global analysis and representation of the results.

The detection of ADRs of interest should be followed by an impact analysis. Several factors can be used to characterize a case of interest. According to the British Medicines & Healthcare Products Regulatory Agency, these factors can be grouped into four categories: Strength, New, Important, Preventive (SNIP) (Graham et al., 2000), i.e., (1) the strength of the signal, (2) the unexpected aspect of the ADR, (3) the seriousness of the reaction, and (4) the possibility to implement preventive measures. Several methods have been proposed to analyze the impact of observed signals (Heeley et al., 2005; Waller et al., 2005; Rolfes et al., 2016).

We do not recommend systematically recording information from posts in a pharmacovigilance database, as the cases are less informative and concern less serious ADRs. Posts related to potential ADRs should be stored in a dedicated database that allows statistical data analysis. Manual review of posts should be restricted to drug safety issues. In this context, forums should only be considered as a complementary source of information for pharmacovigilance. The review of posts appears to be more informative for safety issues that concern young people and women, who are more likely to publish in social media. Exploiting web forums should not be limited to the detection of ADRs or misuse. They may also be useful for studying patient sentiments concerning certain health issues or drugs. This will require the study of both the volume of posts and how patients express their sentiments.

## Conclusion

Safety data from web forums are less informative than in the FPVD, as forum users do not intend to post data in a “pharmacovigilance” format. Nevertheless, the Vigi4med project demonstrated that ADRs described by users of web forums may be useful for pharmacovigilance. This observation justifies considering the extraction of pharmacovigilance data from web forum posts, despite their limited quality.

Moreover, although posts were less informative and concern less serious ADRs than those reported in the FPVD, we found more unexpected ADRs in the forums and their SOCs were different. Thus, web forums should be considered as a complementary source for pharmacovigilance.

## Author Contributions

CB, JL, AB, AL-LL, M-NB, FB, and M-CJ wrote the evaluation protocol. BA extracted data from the web forums. FM-H and CG developed the natural language processing algorithm to detect the mention of drugs and adverse events. DL developed the web form used by FB, RA, M-NB, and AL-LL to evaluate the posts. The web form was designed by M-CJ, CB, AL-LL, M-NB, and FB. PK and SK performed the statistical analysis. PK, AL-LL, FB, JL, and CB wrote the first draft of the manuscript. AB, BA, M-CJ, RA, and CG wrote sections of the manuscript. CB supervised the work and was responsible for submitting the Vigi4Med project proposal to the ANSM with AB, M-CJ, AL-LL, MN–B, and SK. All authors contributed to revising the manuscript and read and approved the submitted version.

## Conflict of Interest Statement

The authors declare that the research was conducted in the absence of any commercial or financial relationships that could be construed as a potential conflict of interest.

## Footnotes

1 http://www.emse.fr/tildezimmermann/Vigi4Med

2 MedDRA terminology is organized into 27 SOCs – highest hierarchical category – and divided into four other levels, including PTs.

3 French acronym for *Agence Nationale de Sécurité du Médicament et des Produits de Santé*

4 In this section, the term significantly is used when *p* < 5%.

## Abbreviations

ADR: adverse drug reaction
AE: adverse event, possibly without a causal relationship with a drug
ANSM: French Agency for Drug Safety (French acronym of *Agence Nationale de Sécurité du Médicament et des Produits de Santé*)
FPVD: French Pharmacovigilance Database
HAS: French National Authority for Health (French acronym of *Haute Autorité de Santé*)
HON: Health On the Net
MedDRA: medical dictionary for regulatory activities
NLP: natural language processing
P: *p*-value
PT: preferred term
PRR: proportional reporting ratio
SOC: system organ class
SPCs: summary of product characteristics
TTO: time-to-onset
TRU: temporary recommendation for use
